# Using multiple mini interviews as a pre-screening tool for medical student candidates completing international health electives

**DOI:** 10.1080/10872981.2018.1483694

**Published:** 2018-06-18

**Authors:** Caley A. Satterfield, Matthew M. Dacso, Premal Patel

**Affiliations:** Center for Global Health Education, University of Texas Medical Branch, Texas, Galveston, US

**Keywords:** Multiple Mini Interviews, MMIs, Global Health Education, International Health Electives, IHEs

## Abstract

There continues to be an increase in the number of learners who participate in international health electives (IHEs). However, not all learners enter IHEs with the same level of knowledge, attitude, and previous experience, which puts undue burden on host supervisors and poses risks to student and patient safety. The Multiple Mini-Interview (MMI) is a technique that has become a popular method for undergraduate and postgraduate-level health science admissions programs. This paper describes the MMI process used by our program to screen first-year medical students applying for pre-clinical IHEs. Two country-specific cases were developed to assess non-cognitive skills. One hundred percent (100%) of the students (n = 48) and interviewers (n = 10) who participated in MMIs completed anonymous surveys on their experience. The majority of students rated the scenarios as realistic (>90%); 96% found the MMI format fair and balanced; 96% of students felt that they were able to clearly articulate their thoughts; 75% of students stated that they had a general understanding of how the MMIs worked; only 33% of students would have preferred a traditional one-to-one interview. Feedback from both interviewers and students was positive toward the MMI experience, and no students were identified as unfit for participation. Ultimately, 43 students participated in pre-clinical IHEs in 2016. In this paper, we will outline our MMI process, detail shortcomings, and discuss our next steps to screen medical students for IHEs.

## Introduction

Health science students throughout the United States and Canada express increasing interest in opportunities to study abroad. Both the number of health science trainees participating in international health elective (IHE) courses and the number of institutions offering these IHE courses continue to rise. As a result, global health programs are called upon to organize formal pre-departure training that addresses, among other topics, the ethics of short-term medical volunteerism. The need for pre-departure training was highlighted as a key aspect of ethical global health training by Sugarman and Crump and the Working Group on Ethics Guidelines for Global Health Training (WEIGHT) [1]. These guidelines promote an ethical approach to IHEs that optimizes both patient and trainee safety. Although pre-departure training for IHEs is far from standardized or mandated for all global health experiences, progress has been made. Many sending institutions and organizations now realize that the need for such training exists [2]. Pre-departure training methods and evaluations are becoming the norm in peer-reviewed publications and presentations at key global health meetings. Repositories of educational materials developed and vetted by leading global health education professionals have also seen growth in recent years [3].

The WEIGHT committee listed the following as an ethical principle to follow when sending students on global health experiences:

*Select trainees who are adaptable, motivated to address global health issues, sensitive to local priorities, willing to listen and learn, whose abilities and experience matches the expectations of the position, and who will be good representatives of their home institution and country* ([1], p. 1190).

At this time, there is no literature that outlines a method to implement this guideline and select trainees with characteristics most suited to participate in IHEs.

Behavioral issues, mismatched expectations, and mental health difficulties place an undue burden on host sites and thus may have a negative impact on global health partnerships and, most importantly, patient care. Conversely, selecting appropriate trainees for specific field sites helps match student expectations with field site capabilities, and decrease conflict points between trainees and their potential supervisors. Appropriate selection should also identify trainees who may need additional remediation prior to travel either for academic or behavioral expectations and to screen students who are ill-suited for international travel and IHEs in general.

### 

#### What are MMIs?

Over the past several years, increasing numbers of undergraduate and postgraduate health science training programs have begun using Multiple Mini-Interviews (MMIs) in their admissions programs. Multiple Mini Interviews (MMIs) are a series of highly structured, focused stations or encounters with separate interviewers or observers [4,5]. During the interview, participants are rated on their skill at deciphering an ethical conflict or problem and articulate a response to the hypothetical situation that reflects elements of emotional intelligence or psychological maturity [4,6]. The purpose of the MMI is not to develop a station where there is a right or wrong answer, but rather to develop stations that can illuminate interviewee skills such as critical thinking, creativity, problem-solving, and personal and social awareness [4,5,7].

#### History of MMIs

Multiple Mini Interviews (MMIs) were first piloted by McMaster University in Ontario, Canada as a new, more valid method of medical school applicant screening [4]. MMIs were designed to elicit details of applicants’ abilities in areas such as interpersonal skills, communication skills, teamwork skills, and professionalism, which are highly valued in the health professions [4]. Traditional interview methods have been found to have poor predictive validity. Furthermore, they are fraught with inter-rater reliability and problems related to the implicit bias of interviewers. Currently, health professions schools in the areas of dentistry, physician’s assistant, pharmacy, paramedicine, nursing, and midwifery; medical schools; and residency programs in North America and the United Kingdom are using the MMI for selecting candidates for their programs [4,6–12].

#### Traditional interviews versus MMIs for admissions decisions

When evaluating for specific attitudes or behaviors, traditional interviews have been shown to have poor inter-rater reliability, susceptible to bias, and outcomes have been found to be related to context-specificity, not the performance of the interviewee [4,6,13–15]. Additionally, traditional interviews are time-intensive and taxing as they can last a wide-ranging timeframe depending on the situation [4,5]. Furthermore, traditional interviews have not been found to have a high predictive value for future success in clinical training, while grade point average (GPA) and other quantitative measures only predict success in the pre-clinical years [14,15].

In contrast, MMIs allow multiple encounters between applicants and many different interviewers, thus reducing bias and allowing students who may perform poorly in one encounter to start fresh in a new encounter [5]. Additionally, MMIs are highly structured, with all applicants encountering the same cases and rating scales [9]. Previous MMI evaluations have suggested that the MMI format produces more valid results and has better predictive value than standard interviews because it allows for a more holistic view of an applicant based on multiple observers [5,6,9]. Reduced time with applicants has been cited as both a pro and con of the MMI format (5–8 min compared to 45-min traditional interviews) [5].

#### UTMB global health MMI

In 2016, we implemented the Multiple Mini Interview (MMI) to select first-year pre-clinical medical students for IHEs based on the guidelines established by WEIGHT. The MMI process was intended to evaluate the suitability of medical students for specific IHEs. Applications soliciting students’ prior experiences and personal statements are also part of this process. Additionally, UTMB faculty mentors and host collaborators review applicants, to place students in IHE experiences most suited to the host’s needs and the desired student experience.

### Objective

The objective of this educational innovation was to develop and pilot a screening process for first-year medical student candidates applying for pre-clinical IHE participation sponsored by the University of Texas Medical Branch.

## Methods

### Ethics approval

The development, implementation, and evaluation of the MMI as a screening process for pre-clinical medical student IHEs, was granted exempt status from the Institutional Review Board as a quality improvement project. Additionally, the Education Research Committee of the School of Medicine Curriculum Committee approved the plan to utilize this data for dissemination.

### Materials

Two MMI case scenarios were developed by a committee of global health faculty based on real-life situations encountered by our program. The case scenarios were developed to specifically assess the following characteristics: flexibility, professionalism, humility, open-mindedness, ability to navigate new situations, cultural awareness, self-awareness, awareness of role and limitations, and safety/risk-taking. These characteristics are in keeping with the recommendations set forth by the WEIGHT. Table 1 outlines the characteristics in each case scenario.

**Table 1.** Case scenario characteristics.

Scenario 1 – Kenya	Scenario 2 – South America
Professionalism	Flexibility
Cultural awareness	Professionalism
Ability to navigate new situations	Humility
Self awareness	Open-mindedness
Safety/risk taking (personal)	Safety (patient)
	Awareness of role/limitations

Interviewer versions of the case scenarios contained interview questions and suggestions for additional probes for situations in which a student did not provide a complete answer. Appendix A and Appendix B contain the two case scenarios with interviewer questions developed for this exercise.

To assess performance, we developed two candidate-rating rubrics that were specific to the desired candidate characteristics under review in each case scenario. Each rubric contained categories on Attitudes and Behavior, though the specific characteristic under those main categories varied by case scenario. The full candidate-rating rubrics for both case scenarios can be found in Appendix C and Appendix D. Written instructions for both students and interviewers were adapted from another UTMB program using MMIs of which the authors were also members. These can be found in Appendices E and F.

Two global health faculty members with previous MMI experience participated in developing cases, rubrics, and training additional interviewers for the process. Interviewers included faculty, staff, and students. A 1-h training session was held with interviewers to explain the MMI process, go over interviewer instructions, review the case scenarios, and answer questions regarding how to use the candidate-rating rubric. Notably, interviewers were selected based on previous experience with the particular challenges of each field site.

### Participants

Forty-eight first-year preclinical medical students participated in the MMI screening process in 2015. Ten interviewers were needed to facilitate the MMIs in the timeframe allotted. Among these 10 interviewers, 6 were faculty members, 2 were staff, and 2 were students (PhD student and senior medical student).

### Process

A mock clinical space for simulation with standardized patients was used for the MMI process. The space proved optimal due to the waiting area, individual rooms with two-way mirrors and audio, public announcement system, and a secondary exit door. The MMI process was after business hours on one day, during a 3-h timeframe. Students received an email with an interview time 6 days prior to the interview date. Second-year medical student volunteers organized students in the waiting room and presented each interviewing student with written instructions at check-in and a starting room number. A staff member utilized the public announcement system to time the encounters and move students to next station. As per previously published MMI formats [14], students were given 2 min to read the scenario and 8 min to discuss the case with the interviewer. Students completed optional anonymous paper-based evaluations upon completion of the two stations (Appendix G). Interviewers completed an anonymous, paper-based evaluation form at the conclusion of the interview session (Appendix H).

### Data analysis

Both student and faculty evaluation data were entered verbatim into an online survey system to generate an excel document of results [16].

#### Students

Likert-type data were converted from word-based scales to numeric data for analysis with 1 = strongly disagree, 2 = disagree, 3 = agree, and 4 = strongly agree. Quantitative data were analyzed using IBM SPSS Statistics 23 to determine response frequency. Thematic analysis was used to analyze qualitative feedback [17].

#### Interviewers

Likert-type quantitative data, with 4 = Very much, 3, 2, and 1 = Not at all, were analyzed using IBM SPSS Statistics 23 to determine response frequency, while qualitative feedback was based on themes [17].

## Results

### Students

Scoring of the MMI for student selection was based on the total score of both rubrics (24 possible points). Table 2 shows the results of the scored rubrics, individually and combined.

**Table 2.** Individual and combined rubric results.

Rubric	Mean	SD	95% CI
Rubric 1 (12 points)	11.44	.79	−1.96, 1.96
Rubric 2 (12 points)	11.65	.70	−1.96, 1.96
Combined (24 points)	23.08	.96	−1.96, 1.96

Student MMI participant responses were positive about the experiences. Table 3 shows the response frequency for each of the Likert-type questions evaluated about the general MMI experience. Table 4 shows the response frequency for questions related to the scenarios.

**Table 3.** General MMI experience (frequency).

Question M (SD)	Strongly agree % (F)	Agree % (F)	Disagree % (F)	Strongly disagree % (F)
Format stressful 2.02 (.729)	2.1% (1)	20.8% (10)	54.2% (26)	22.9% (11)
Able to articulate myself 3.23 (.555)	29.2% (14)	64.6% (31)	6.3% (3)	0% (0)
Understood format 3.06 (.909)	37.5% (18)	37.5% (18)	18.8% (9)	6.3% (3)
Fair and balanced 3.44 (.580)	47.9% (23)	47.9% (23)	4.2% (2)	0% (0)
Interviewers listened 3.71 (.459)	70.8% (34)	29.2% (14)	(0%) 0	0% (0)
More talking than interviewer 3.71 (.504)	72.9% (35)	25% (12)	2.1% (1)	0% (0)
Prefer standard interview 2.25 (.583)	4.2% (2)	29.2% (14)	54.2% (26)	12.5% (6)

**Table 4.** MMI scenarios frequency table (student responses).

Question: Kenya M (SD) South America M (SD)	Strongly agree %(F)	Agree %(F)	Disagree %(F)	Strongly disagree %(F)
Realistic scenario				
3.58 (.577)	62.5% (30)	33.3% (16)	4.2% (2)	0% (0)
3.50 (.583)	54.2% (26)	41.7% (20)	4.2% (2)	0% (0)
Understood scenario				
3.58 (.539)	60.4% (29)	37.5% (18)	2.1% (1)	0% (0)
3.58 (.577)	62.5% (30)	33.3% (16)	4.2% (2)	0% (0)
Easy to talk about				
3.25 (.668)	37.5% (18)	50% (24)	12.5% (6)	0% (0)
3.65 (.483)	64.6% (31)	35.4% (17)	0% (0)	0% (0)
Clear ethical dilemma				
3.60 (.536)	62.5% (30)	35.4% (17)	2.1% (1)	0% (0)
3.77 (.425)	77.1% (37)	22.9% (11)	0% (0)	0% (0)

Qualitative feedback provided insight into areas that may need to be clarified or revised for future iterations of the MMI project as a screening method for global health experiences. Thematic analysis of the qualitative feedback resulted in five themes. A theme was derived for three or more occurrences in the student evaluation. The themes found are summarized as follows:

## Thematic analysis (frequency of occurrence in student evaluation)

- Good experience (9)
- Need more time (4)
- Prefer a standard interview, in addition or in lieu of MMI (3)
- Realistic case situations (3)

### Interviewers

Interviewer responses were also positive about the experience, and they also provided constructive feedback on ways to improve the MMI process to better select global health experience participants. Most interviewers had not participated in a MMI prior to this encounter. Table 5 shows the response frequency for each of the Likert-type questions evaluated about the general interview process. Table 6 shows the response frequency for the Likert-type questions related to the specific scenarios. Qualitative feedback varied greatly by each interviewer, with the following four suggestions/dilemmas representing the cumulative response:

- Need more time with students (15 min suggested)
- Greater flexibility with probing questions/ability to deviate from script/better probing questions
- Scenarios did not provide a way to distinguish much between students
- Students understand the importance of rule-following but lack of understanding or do no articulate understanding of importance of social/community relationships

**Table 5.** General MMI interviewer experience frequency.

Question M (SD)	4 = very much	3	2	1 = not at all
Able to differentiate students 3.20 (.789)	40% (4)	40% (4)	20% (2)	0% (0)
Enough time 3.70 (.675)	80% (8)	10% (1)	10% (1)	0% (0)
Ranking form was clear 3.90 (.316)	90% (9)	10% (1)	0% (0)	0% (0)
Ok with no ‘neutral’ or ‘average’ ranking 3.90 (.316)	90% (9)	10% (1)	0% (0)	0% (0)

**Table 6.** MMI scenarios frequency interviewers.

Question: Kenya M (SD) South America M (SD)	4 = very much	3	2	1 = not at all
Easy for students to talk about				
4.00 (.000)	100% (5)	0% (0)	0% (0)	0% (0)
4.00 (.000)	100% (5)	0% (0)	0% (0)	0% (0)
Scenario was realistic				
4.00 (.000)	100% (5)	0% (0)	0% (0)	0% (0)
4.00 (.000)	100% (5)	0% (0)	0% (0)	0% (0)
Scenario caused students to weigh alternatives				
2.80 (.837)	20% (1)	40% (2)	40% (2)	0% (0)
3.40 (.548)	40% (2)	60% (3)	0% (0)	0% (0)

## Discussion

Screening IHE applicants for knowledge, attitude, and behavior characteristics is supported by consensus-based guidelines [1]. Such a process should aim to match the most appropriate students with the most suitable field site based on host institution feedback. This process should also reduce disciplinary and professionalism issues. Our use of the MMI format to screen medical students for IHEs allowed interviewers to have personal contact with each applicant and listen to student responses to case scenarios involving real world ethical dilemmas. Both students and interviewers rated the MMI experience highly.

The MMI process ultimately presented some limitations in our screening ability. Interviewers felt constrained by the rigid structure, which prevented them from being able to delve into topics in greater depth. The limited range of interview scores (21–24) highlights the lack of differentiation among students. We might attribute the lack of differentiation to the homogeneity of our student applicants; or perhaps the MMI assessment was not designed to provide a wider distribution of scores. Interviewers felt the scenarios, while real world examples, allowed students to provide canned responses that did not reflect deeper thought about the downstream consequences of their actions on the community, partnership, or patient safety. This may have contributed to the lack of differentiation. Students felt that they did not have the time or the opportunity to discuss topics more deeply or to communicate why they wanted to participate in an IHE at a particular field site. Recently, it has been suggested that generalizability is an inherent limitation of MMIs due to the need to customize MMIs to the specific content and context needed [18]. We see this as also a benefit of the MMI, allowing global health programs to develop MMI screenings that match program philosophy and learner type.

### 

#### Lessons learned and possible solutions

**Table:** 

Lesson learned	Possible solution
● Cases too straightforward	● Restructure cases with multiple defensible courses of actions ● More probing questions with fewer limitations ● More time for interviews
● Not enough time	● Allocate more time
● Multiple issues per scenario	● Develop more scenarios with fewer ethical dilemmas per scenario

### Next steps

The MMI experiment was an important first step in improving our student selection process for IHEs to align with established ethical guidelines for IHEs. Selecting appropriate students for global health fieldwork remains a high priority. For the 2017 preclinical preceptorship period, we have built upon our previous experience to deploy a new interview strategy that combines elements of the MMI with structured panel interviews. The structured panel interview will be added to help differentiate students. The interview panels will be composed of the UTMB mentor for each field site or field site region, a second global health educator, and a previous student selected by the UTMB mentor. Students will be assigned to a panel based on a pre-screening of their ranked field site preferences.

Future studies should explore the long-term impact of student selection on host site satisfaction, community satisfaction, and clinical or research productivity as appropriate for the rotation type. Additionally, future evaluations of selection methods for IHEs should include a plan for reporting and documenting professionalism and behavioral issues.

### Conclusion

Selecting students for global health field experience is a complicated process. There is scant literature that describes best practices for selecting students for IHEs. Very few published papers address desired characteristics of global health student participants [1]. Pre-departure training, although not standardized, is now a focus for academic institutions that send health professional students abroad for global health education experiences. To ethically engage international partners in limited-resource settings with students, it is imperative to develop best practices for selecting students who possess characteristics that will position them to add value during international fieldwork. Some behaviors and characteristics, such as flexibility, professionalism, empathy, and ethical understanding are crucial for successful global health fieldwork. These factors are notoriously difficult to instill through pre-departure training [1,8,10,19–24]. This pilot evaluation leads to a better understanding of what is needed for an effective screening process. We hope that our experience will inform other global health programs that want to bring their student selection processes in line with the most current ethical guidelines.

## Appendix A

**Scenario 1 – Kenya**

You are a first-year medical student at a mission hospital in Kenya. You have been told that there is a code of conduct and some expectations for all students such as a curfew at 8 pm, no drinking alcohol, and communal meals. However, local Kenyan students tell you that these rules are not heavily enforced and that sometimes they have parties with alcohol.

You have become close friends with some of the final year Kenyan medical students (interns), and it is your last week in Kenya. The interns have invited you to a party located 20 km from the hospital campus where you are staying. All of the UTMB students have been invited. In Kenya, it is a great honor to be invited to celebrations, and you do not want to hurt their feelings by turning down the invitation.

As you are getting ready to leave, you notice that all of the Kenyan students are taking moto-taxis (motorcycles) to the party. It is a common mode of transportation in Kenya and involves riding on a motorcycle without a helmet and with several passengers. UTMB students are supposed to use the private transportation service, but it is three times the cost and you are running low on funds.

When you arrive at the party, you realize that alcohol is being served. Questions:

- What are the important issues in this scenario?
- What are the dilemmas regarding transportation? What would you do and why?
- What are the dilemmas regarding alcohol at the party? What would you do and why?

As a UTMB student, you are an Ambassador for the UTMB Global Health program. How does this make your choices in Kenya different than those you would make at home?

## Appendix B

**Scenario 2 – South America**

You are a first-year medical student participating in a global health preclinical elective in South America. Before arrival, you were preparing to do a research project focused on emerging infectious disease epidemiology, which would give you some time in the lab and in the field collecting data. Two weeks into your rotation the IRB has still not been approved and your UTMB mentor and your host mentor are working on preparing an alternative project for you. In the meantime, you are told to accompany the medical teaching service on rounds in the hospital.

During your first day of rounds, many of the patients you see are presenting with chronic cough and fever and are being evaluated for Tuberculosis, a disease that you know from your PHD course to be highly contagious. You have an N95 mask in your pocket, but notice that no one else seems to be wearing them.

After rounds, the resident tells you to come with her to see ‘a really interesting case.’ When you arrive at the bedside you see a 16 year-old girl who is confused and complaining of headache. The resident tells you that she thinks this patient has TB Meningitis. He introduces you to the mother and tells her ‘this doctor will do the LP.’

Questions:

- What are the important issues in this scenario?
- How would you respond to your project falling through?
- What are the safety concerns in this scenario? (if the student does not mention patient safety, you may prompt him/her)
- What would you do in this situation?

Describe how you can decline the LP. What would you say and how?

## Appendix C

Global Health MMI Score Sheet – Scenario 1: Kenya

Attitude

(**1) Cultural Awareness**

**Table:**

1	2	3
Demonstrates insensitivity to the social norms held by host community.	Naïve understanding of social norms.	Demonstrates sensitivity to the social norms held by host community.

(**2) Awareness of Role/Limitations (Social)**

**Table:**

1	2	3
Unable to recognize appropriate role for UTMB medical student in this situation. Fails to understand appropriate limitations.	Recognizes limitations but cannot articulate appropriate role of UTMB medical student.	Recognizes role of UTMB medical student in this situation and sets appropriate limitations.

Behavior

(**1) Personal Safety and Risk-taking**

**Table:**

1	2	3
Unaware of personal safety and risks involved in scenario.	Recognizes personal safety issues and risks, but cannot articulate acceptable practices to protect personal safety.	Demonstrates awareness of personal safety issues and risks. Articulates acceptable practices to protect personal safety.

**ON BACK**

(**1) Conduct**

**Table:**

1	2	3
Demonstrates disregard for policy or demonstrates willingness to break policy.	Recognizes policy restrictions but advocates for policy exceptions.	Adheres to policy and advocates the need to follow policy.

_____This student needs one-to-one remediation before being placed at a field site. Why?

________________________________________________

This student should NOT be placed at a field site.

Why?

________________________________________________

Notes:

________________________________________________

________________________________________________

________________________________________________

________________________________________________

________________________________________________

## Appendix D

Global Health MMI Score Sheet – Scenario 2: South America

Attitude

(**1) Flexibility/Open-mindedness**

**Table:**

1	2	3
Unable or unwilling to adapt to changing situation. Greets changing situation with anger, defiance, and/or negativity.	Expresses lukewarm emotion toward change, but can understand necessity for changing circumstances.	Shows acceptance for the instability of global health fieldwork with positive attitude. Expresses flexibility and openness to new project ideas and/or new situations. May still express disappointment at change of project.

(**2) Awareness of Role/Limitations (Clinical Experience)**

**Table:**

1	2	3
Unable to recognize appropriate role for preclinical medical student in clinical situations. Fails to understand appropriate limitations.	Recognizes limitations but cannot articulate appropriate role.	Recognizes role of preclinical students in clinical situations and sets appropriate limitations.

Behavior

(**1) Personal Safety and Risk-taking**

**Table:**

1	2	3
Unaware of personal safety and risks involved in scenario.	Recognizes personal safety issues and risks, but cannot articulate acceptable practices to protect personal safety.	Demonstrates awareness of personal safety issues and risks. Articulates acceptable practices to protect personal safety.

**ON BACK**

(**2) Patient Safety**

**Table:**

1	2	3
Unable to identify patient safety issues involved in scenario and/or they promote inappropriate practices.	Recognizes patient safety issues, but cannot articulate acceptable practices to protect patient safety.	Demonstrates awareness of patient safety issues. Articulates acceptable practices to protect patient safety.

_____This student needs one-to-one remediation before being placed at a field site. Why?

________________________________________________

This student should NOT be placed at a field site.

Why?

________________________________________________

Notes:

________________________________________________

________________________________________________

________________________________________________

________________________________________________

________________________________________________

## Appendix E

**Global Health MMI Information for Students**

There are two cases. Below are instructions for how the Global Health MMI process will work. You will spend approximately 30 min completing both interview sessions and an additional 5 min completing the feedback and evaluation form.

**Rotating Rooms**: If you are assigned to start in room 1 you will rotate to room 2 for your second case. Room 2 goes to 3, etc.…room 10 goes to 1.

*Instructions*

- Students will be given 2 min to read the scenario posted outside the room. Do NOT start reading until instructed to do so.
- When instructed, enter your interview room. Once you enter your room you will have 8 min to respond to the scenario (a copy will also be in the room for you).
- You will get a 1-min warning.
- At the conclusion of the first interview exit the room and wait outside the door (a chair is available. You will wait an additional 3 min.
- When instructed, move to your next interview room.
- Students will be given 2 min to read the scenario posted outside the room. Do NOT start reading until instructed to do so.
- When instructed, enter your interview room. Once you enter your room you will have 8 min to respond to the scenario (a copy will also be in the room for you).
- You will get a 1-min warning.
- After the second interview, exit and complete the feedback evaluation and return to the labeled box near Brad Brock, who will be stationed in the center of the SP center near the exit.

*Helpful Information*:

- If you finish the interview early, you can exit the room. There is nothing wrong with a short interview.
- Your interviewer will take notes during your interview. Do not worry about this!
- If you have questions about field sites or projects, or anything else related to your global health rotation, please email Caley Satterfield at casatter@utmb.edu for an appointment. There will not be time to address questions during the MMI process.

Acknowledgement: Instructions adapted from UTMB Student Continuity of Practice Experience (SCOPE) MMI instructions, provided by author Dr. Premal Patel.

## Appendix F

**MMI Instructions for Interviewers**

- Students will be given 2 min to read the scenario posted outside the room.
  - Once they enter your room they will have 8 min to respond to the scenario (a copy will also be in the room for them).
- When the students enter the room they will give you a rubric with their name on it. Please remember that these are first-year medical students in training and maturity and rate them accordingly.
- If you feel strongly that a student needs one-to-one remediation before being placed at a field site, please mark the appropriate box on the rubric and briefly explain why.
- If you feel strongly a student is not suited for global health fieldwork, please indicate by checking the box on the rubric and briefly explaining why you feel this student should not be placed at a field site.
- Students are not required to wear a white coat, but they should dress professionally. Please note if they are not dressed appropriately.
- Students will have 8 min in the room with you, with a 1-min warning. You will have an additional 3 min when the student leaves the room to take notes and mark the rubric.
- Use the prompts on the scenario provided to you as needed.
- Try to rate as you go, since you would not have much of a break between students.
- Be sure to put your name on the rating sheet as the interviewer.

Acknowledgement: Instructions adapted from UTMB Student Continuity of Practice Experience (SCOPE) MMI instructions, provided by author Dr. Premal Patel.

## Appendix G

MMI Student Feedback

**Questions**

## Appendix H

MMI Faculty Feedback**Questions**
